# Diversity and characteristics of culturable endophytic bacteria from *Passiflora edulis* seeds

**DOI:** 10.1002/mbo3.1226

**Published:** 2021-08-15

**Authors:** Aoi Ishida, Toshiki Furuya

**Affiliations:** ^1^ Department of Applied Biological Science Faculty of Science and Technology Tokyo University of Science Noda Chiba Japan

**Keywords:** biocatalysis, endophyte, passion fruit, piceatannol, resveratrol, seed endophyte

## Abstract

Defense compounds generally inhibit microbial colonization of plants. In this study, we examined the presence of endophytes in *Passiflora edulis* seeds that accumulate resveratrol and piceatannol at extremely high levels as defense compounds. Interestingly, although no microbial colonies appeared on an agar growth medium from the cut or homogenized seeds, colonies were generated from cut seedlings derived from the seeds. A total of 19 bacterial strains were isolated, of which 15 were classified as Gram‐positive. As we hypothesized that extremely high levels of piceatannol in the seeds would inhibit the growth of endophytes cultured directly from the seeds, we examined the antimicrobial activity of this compound against the isolated bacteria. Piceatannol exerted bacteriostatic rather than bactericidal effects on most of the bacteria tested. These results suggest that the bacteria remain static in the seeds due to the presence of piceatannol and are transmitted to the seedlings during the germination process, enabling colonies to be established from the seedlings on the agar medium. We also investigated the biocatalytic activity of the isolated bacteria toward resveratrol and piceatannol. One bacterium, *Brevibacterium* sp. PE28‐2, converted resveratrol and piceatannol to their respective derivatives. This strain is the first endophyte shown to exhibit such activity.

## Graphical abstract

BLURB FOR ETOC: Bacterial endophytes were isolated from Passiflora edulis seeds that accumulate defense compounds at extremely high levels. Although no microbial colonies appeared on the agar growth medium from the cut or homogenized seeds, colonies were generated from cut seedlings derived from the seeds. The defense compound piceatannol exerted bacteriostatic rather than bactericidal effects on most of the isolated bacteria.

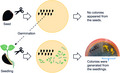

## 1 INTRODUCTION

*Passiflora edulis* is a plant in the passion flower family (Passifloraceae). The fruit of this plant is called passion fruit, and it is widely consumed in its natural state, with the seeds, or processed as a tropical juice. Interestingly, *P*. *edulis* seeds accumulate stilbene derivatives as secondary metabolites at very high levels compared with other plant tissues. For example, the seeds are rich in resveratrol (3,5,4′‐trihydroxy‐*trans*‐stilbene, 0.1 mg/g) and piceatannol (3,4,3′,5′‐tetrahydroxy‐*trans*‐stilbene, 2.2 mg/g), with the latter containing at markedly higher levels (Matsui et al., 2010; Piotrowska et al., 2012). These secondary metabolites function as defense compounds that protect the plant tissues from the extensive damage that can be caused by microbes. In addition to exhibiting antimicrobial activity, these compounds have various health‐benefiting properties. For example, resveratrol exhibits antioxidant, anticancer, and anti‐inflammatory activities. Piceatannol has biological activities similar to those of resveratrol. Furthermore, piceatannol exerts positive effects on human dermal cells by inhibiting melanogenesis and promoting collagen synthesis, and these effects are more pronounced than those of resveratrol (Matsui et al., 2010; Seyed et al., 2016). Other stilbene derivatives also reportedly exhibit a variety of biological activities (Jeandet et al., 2021).

Endophytes are bacteria or fungi that colonize the interior parts of plants without harming the host. They have been found in almost every plant species examined to date (Liu et al., 2017; Reinhold‐Hurek & Hurek, 2011). Endophytes primarily inhabit the roots, stems, and leaves. However, only a few reports to date have described the isolation of microorganisms from *P*. *edulis*. A fungal isolate from *P*. *edulis* leaves, which was identified as *Phyllosticta* sp., produced antibacterial metabolites (Santos et al., 2017). In another study, a strain of the fungus *Phialemonium curvatum* was isolated from the leaves of *P*. *edulis* (Rathnayake et al., 2018). In addition, growing evidence indicates that endophytes also reside within plant seeds (Frank et al., 2017; Shahzad et al., 2018; Truyens et al., 2015). However, the presence of high levels of stilbene derivatives as defense compounds would generally inhibit microbial colonization of the seeds of *P*. *edulis*. As endophytes must adapt to the plant environment, organisms residing within the seeds of *P*. *edulis* would have to be resistant to these antimicrobial compounds.

Considerable research has established that endophytes adapting to an environment rich in biologically active compounds often exhibit biocatalytic activities related to the metabolism of these compounds (Brader et al., 2014; Rodriguez et al., 2016). One of the most studied biologically active compounds isolated from endophytes is paclitaxel, which is used as an anticancer drug. Many paclitaxel‐producing fungi have been isolated from plants of the genus *Taxus* (Zhou et al., 2010). A recent report indicated that *Ovatospora brasiliensis*, isolated from the calebin‐A‐producing plant *Curcuma caesia*, converts curcumin into calebin‐A (Majeed et al., 2019). Based on their biocatalytic potential, endophytes within *P*. *edulis* seeds that accumulate resveratrol and piceatannol might be able to convert these compounds to other valuable stilbene derivatives.

We report here for the first time the isolation of endophytes in *P*. *edulis* seeds. Interestingly, although no microbial colonies appeared on the agar growth medium from the cut or homogenized seeds, colonies were generated from cut seedlings derived from the seeds. A total of 19 bacterial strains were isolated, of which 15 were classified as Gram‐positive. As we hypothesized that extremely high levels of piceatannol in the seeds would inhibit the growth of endophytes cultured directly from the seeds, we examined the antimicrobial activity of this compound against the isolated bacteria. We also investigated the biocatalytic activities of the isolated bacteria toward resveratrol and piceatannol.

## 2 MATERIALS AND METHODS

### 2.1 Isolation and identification of endophytes in *P. edulis* seeds

**FIGURE 1:**
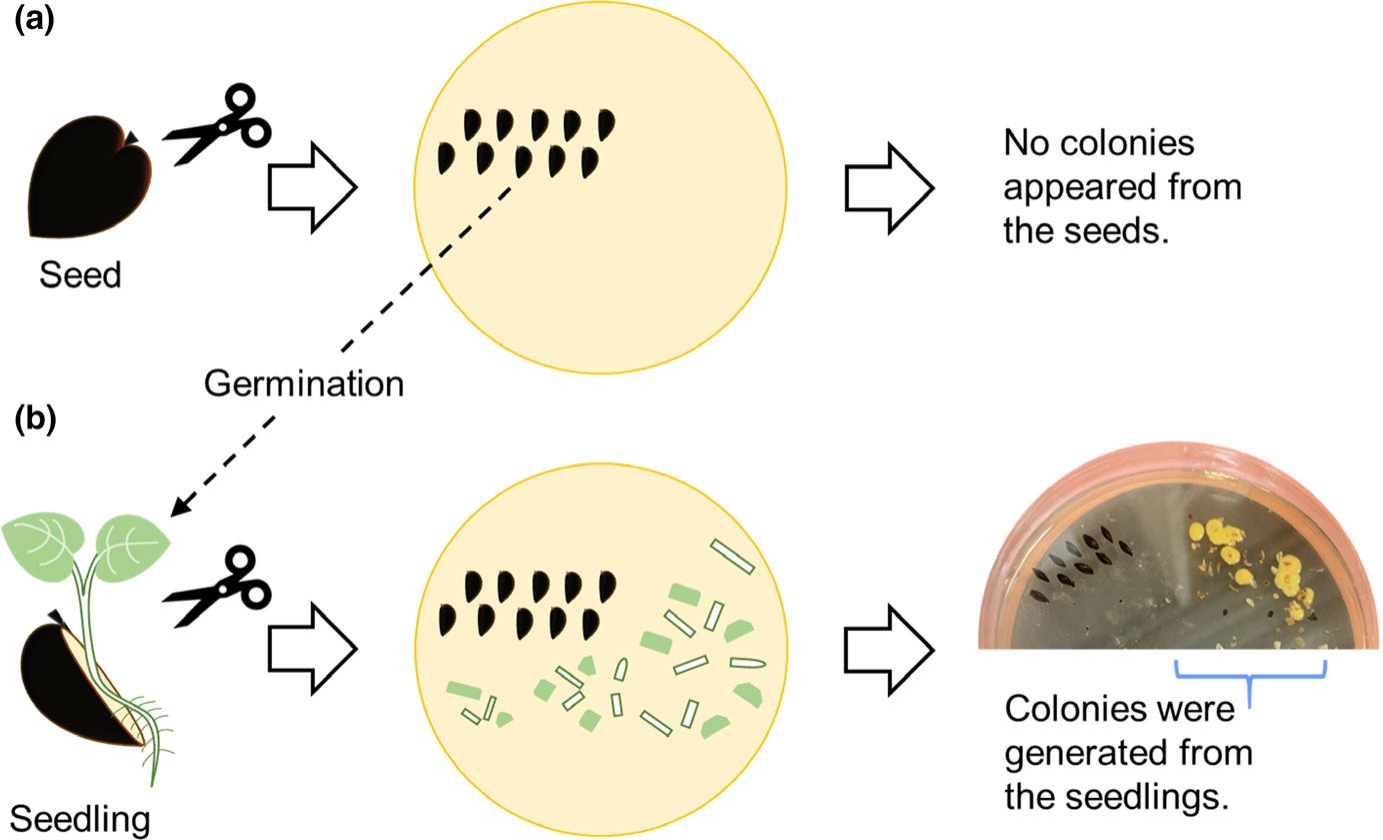
Schematic illustration of the procedure for isolating endophytes from *P*. *edulis* seeds. No microbial colonies appeared on the agar growth medium from cut seeds (a), but colonies were generated from cut seedlings derived from the seeds (b)

Seeds were collected from the fruit of *P*. *edulis* grown in Okinawa and Kumamoto Prefectures, Japan. The seeds were surface‐sterilized by dipping in 5% sodium hypochlorite for 30 min, followed by dipping twice in 70% ethanol for 10 min, after which they were thoroughly rinsed with sterile water, according to previous reports (Kurokawa et al., 2021; Mano et al., 2007; Santos et al., 2017), with some modifications. Each seed was cut and placed onto NBRC802, ISP2, PSA, or PF agar medium and incubated at 30ºC for approximately 1 month. In another way, each seed was homogenized in sterile water using a mortar and pestle and plated onto each agar medium. However, no colonies appeared on the plates. As we observed that the cut seeds sprouted after incubation, the resulting seedlings were aseptically sectioned into small fragments and further incubated on the plates (Figure 1). Colonies were generated from these seedlings under sterile conditions. Single‐colony isolation was repeated for colonies formed around the fragmented seedlings. NBRC802 medium (medium no. 802 used in Biological Resource Center, NITE) contained (per liter) Hipolypepton (10 g), Bacto yeast extract (2 g), MgSO_4_·7H_2_O (1 g), and agar (15 g) (pH 7.0). International *Streptomyces* Project‐2 (ISP2) medium contained (per liter) Bacto yeast extract (4 g), Bacto malt extract (10 g), glucose (4 g), and agar (20 g) (pH 7.0). Potato sucrose agar (PSA) medium contained (per liter) potato (200 g), sucrose (20 g), and agar (20 g) (pH 5.6). Passion fruit (PF) medium contained (per liter) juice made from passion fruit (250 g) and agar (15 g) (pH 7.0).

Isolated bacteria were taxonomically identified based on the 16S rDNA sequence. DNA was amplified from colonies by polymerase chain reaction (PCR) using two oligonucleotide primers, 9F 5′‐GAGTTTGATCCTGGCTCAG‐3′, and 1541R 5′‐AAGGAGGTGATCCAGCC‐3′. PCR was performed using KOD FX Neo polymerase (Toyobo, Osaka, Japan) according to the manufacturer’s recommendations under the following conditions: 94ºC for 2 min, followed by 45 cycles of 98ºC for 10 s and 68ºC for 2 min. After purification, the amplified DNAs were sequenced by Eurofins (Tokyo, Japan). The sequence of the 5′‐terminal region (ca. 500 bp) of each DNA was determined for all strains. The sequences were then compared to those in the GenBank database using BLASTN (https://blast.ncbi.nlm.nih.gov/Blast.cgi. MEGA software (https://www.megasoftware.net/ was used to align the sequences and construct a neighbor‐joining phylogenetic tree (Kurokawa et al., 2021). The nucleotide sequences of the 16S rDNA regions of the isolated bacteria were submitted to GenBank under assigned accession numbers (Table 1).

**TABLE 1:** Bacterial strains recovered from the emerging seedlings from *P*. *edulis* seeds

Strain^a^	Medium	Phylum	Accession no.	Closest type strain (accession no.)	Similarity (%)
PE11‐1	NBRC802	*Actinobacteria*	LC603193	*Janibacter limosus* DSM 11140T (NR_026362.1)	849/876 (97)
PE11‐3	NBRC802	*Actinobacteria*	LC603194	*Dermacoccus nishinomiyaensi*s DSM 20448T (NR_044872.1)	864/882 (98)
PE11‐4	NBRC802	*Actinobacteria*	LC603195	*Dermacoccus nishinomiyaensi*s DSM 20448T (NR_044872.1)	905/924 (98)
PE11‐5	NBRC802	*Actinobacteria*	LC603196	*Rhodococcus corynebacterioides* DSM 20151T (NR_119107.1)	469/473 (99)
PE11‐6	PSA	*Actinobacteria*	LC603197	*Dermacoccus nishinomiyaensis* DSM 20448T (NR_044872.1)	680/697 (98)
PE28‐1	NBRC802	*Actinobacteria*	LC603198	*Microbacterium testaceum* DSM 20166T (NR_026163.1)	652/664 (98)
PE28‐2	NBRC802	*Actinobacteria*	LC603199	*Brevibacterium casei* DSM 20657T (NR_041996.1)	513/519 (99)
PE28‐3	NBRC802	*Actinobacteria*	LC603200	*Microbacterium trichothecenolyticum* DSM 8608T (NR_044937.1)	557/570 (98)
PE28‐4	NBRC802	*Actinobacteria*	LC603201	*Micrococcus luteus* DSM 20030T (NR_037113.1)	880/893 (99)
PE28‐5	NBRC802	*Deinococcus‐Thermus*	LC603202	*Deinococcus radiodurans* DSM 20539T (NR_026401.1)	821/878 (94)
PE28‐6	NBRC802	*Deinococcus‐Thermus*	LC603203	*Deinococcus radiodurans* DSM 20539T (NR_026401.1)	477/494 (97)
PE28‐7	ISP2	*Proteobacteria*	LC603204	*Moraxella osloensis* DSM 6998T (NR_113392.1)	485/488 (99)
PE28‐8	ISP2	*Actinobacteria*	LC603205	*Microbacterium testaceum* DSM 20166T (NR_026163.1)	902/916 (98)
PE28‐9	ISP2	*Proteobacteria*	LC603206	*Sphingomonas aquatilis* DSM 15581T (NR_113867.1)	791/807 (98)
PE28‐10	ISP2	*Actinobacteria*	LC603207	*Microbacterium trichothecenolyticum* DSM 8608T (NR_044937.1)	534/546 (98)
PE28‐11	PSA	*Proteobacteria*	LC603208	*Sphingomonas aquatilis* DSM 15581T (NR_113867.1)	870/885 (98)
PE28‐12	PSA	*Proteobacteria*	LC603209	*Sphingomonas aquatilis* DSM 15581T (NR_113867.1)	579/591 (98)
PE28‐13	PSA	*Actinobacteria*	LC603210	*Microbacterium trichothecenolyticum* DSM 8608T (NR_044937.1)	593/606 (98)
PE28‐14	PF	*Firmicutes*	LC603211	*Bacillus megaterium* DSM 32T (NR_118962.1)	671/673 (99)

^a^ PE11 and PE28 strains were derived from the seeds of *P*. *edulis* grown in Okinawa and Kumamoto prefectures, respectively, Japan.

### 2.2 Evaluation of the antimicrobial activity of piceatannol for the isolated bacteria

The antibacterial activity was measured based on the standard methods of the Clinical and Laboratory Standards Institute (CLSI) (Kusakabe et al., 2019). Isolated bacteria were cultivated at 30ºC for 2 days with reciprocal shaking in test tubes containing 2 mL of LB medium, which contained (per liter) Bacto tryptone (10 g), Bacto yeast extract (5 g), and NaCl (10 g) (pH 7.0). The bacterial culture was adjusted to an OD_600_ of 0.01 using LB medium, and an aliquot of the diluted culture (10 µL) was inoculated into LB medium (1 mL) containing piceatannol (0–1.0 mM) and DMSO (1% [v/v]) and further cultivated at 30ºC for 2 days. After cultivation, the minimal inhibitory concentration (MIC) was determined as the lowest concentration at which no growth was observed by visual inspection and measurement of the OD_600_. Following the determination of the MIC, the minimal bactericidal concentration (MBC) was determined by transferring a 25 µL aliquot from each of the test tubes at the concentration corresponding to the MIC and concentrations above the MIC onto LB agar medium. The plates were incubated at 30ºC for 2 days. The presence or absence of bacterial growth was determined by visual inspection. The MBC was defined as the lowest concentration of the compound at which no growth occurred.

### 2.3 Growth of the isolated bacteria on different carbon sources

Isolated bacteria were cultivated in KG medium (Furuya et al., 2011, 2019), which contained (per liter) (NH_4_)_2_SO_4_ (3 g), KH_2_PO_4_ (1.4 g), Na_2_HPO_4_ (2.1 g), MgSO_4_·7H_2_O (0.2 g), FeCl_2_·5H_2_O (10.6 mg), CaCl_2_·2H_2_O (8 mg), ZnSO_4_·7H_2_O (4 mg), MnCl_2_·4H_2_O (2 mg), CuSO_4_·5H_2_O (0.02 mg), KI (0.2 mg), Na_2_MoO_4_·2H_2_O (0.2 mg), CoCl_2_·6H_2_O (0.2 mg), H_3_BO_3_ (0.4 mg), and NaCl (10 mg) (pH 7.2). The medium was supplemented with resveratrol (1 mM), piceatannol (1 mM), glucose (5 mM), or 4‐hydroxyphenylacetic acid (5 mM) as a carbon source, and dimethyl sulfoxide (DMSO, 1% [v/v]) was used to suspend the compounds. Bacteria were cultivated at 30ºC for 7 days with reciprocal shaking in test tubes containing 4 mL of medium. Bacterial growth was determined by measuring the OD_600_.

### 2.4 Biocatalytic activity of the isolated bacteria toward resveratrol and piceatannol

Isolated bacteria cultivated in KG medium supplemented with glucose or 4‐hydroxyphenylacetic acid were harvested by centrifugation, washed with potassium phosphate buffer (50 mM, pH 7.5) containing glycerol (10% [v/v]), and used for whole‐cell reactions. The reaction mixture (250 μL) contained bacteria (10–30 mg wet cell weight/mL), resveratrol (5 mM) or piceatannol (2 mM), DMSO (2% [v/v]), Tween 80 (1% [v/v]), and potassium phosphate buffer (200 mM, pH 7.5) containing glycerol (10% [v/v]). Reactions were carried out at 30ºC for 24 h with vigorous shaking.

High‐performance liquid chromatography (HPLC) analysis was performed to detect the reaction products using an LC‐20 system (Shimadzu, Kyoto, Japan) with an XTerra MS C18 IS column (4.6×20 mm; particle size, 3.5 μm; Waters, Milford, MA, USA). The post‐reaction mixture was acidified by the addition of HCl (pH 2–3), and methanol (250 μL) was then added. The solution was vigorously shaken and centrifuged, and the resulting supernatant (10 μL) was injected into the HPLC system. Mobile phases A and B were water with 0.1% formic acid and methanol, respectively. A gradient of mobile phase B was programmed as follows: start ratio of 5%, hold at 5% for 3 min, increase to 40% for 1 min, increase linearly to 60% for 10 min, increase to 100% for 1 min, and hold at 100% for 3 min. The flow rate was 0.5 mL min^−1^. Compounds were detected spectrophotometrically at a wavelength of 220 nm. Liquid chromatography‐mass spectrometry (LC‐MS) analysis was performed using a JMS‐T100CS TOFMS (JEOL) with electrospray ionization, as described previously (Furuya & Kino, 2014; Hashimoto et al., 2019).

## 3 RESULTS

### 3.1 Isolation of endophytes in *P. edulis* seeds

**FIGURE 2:**
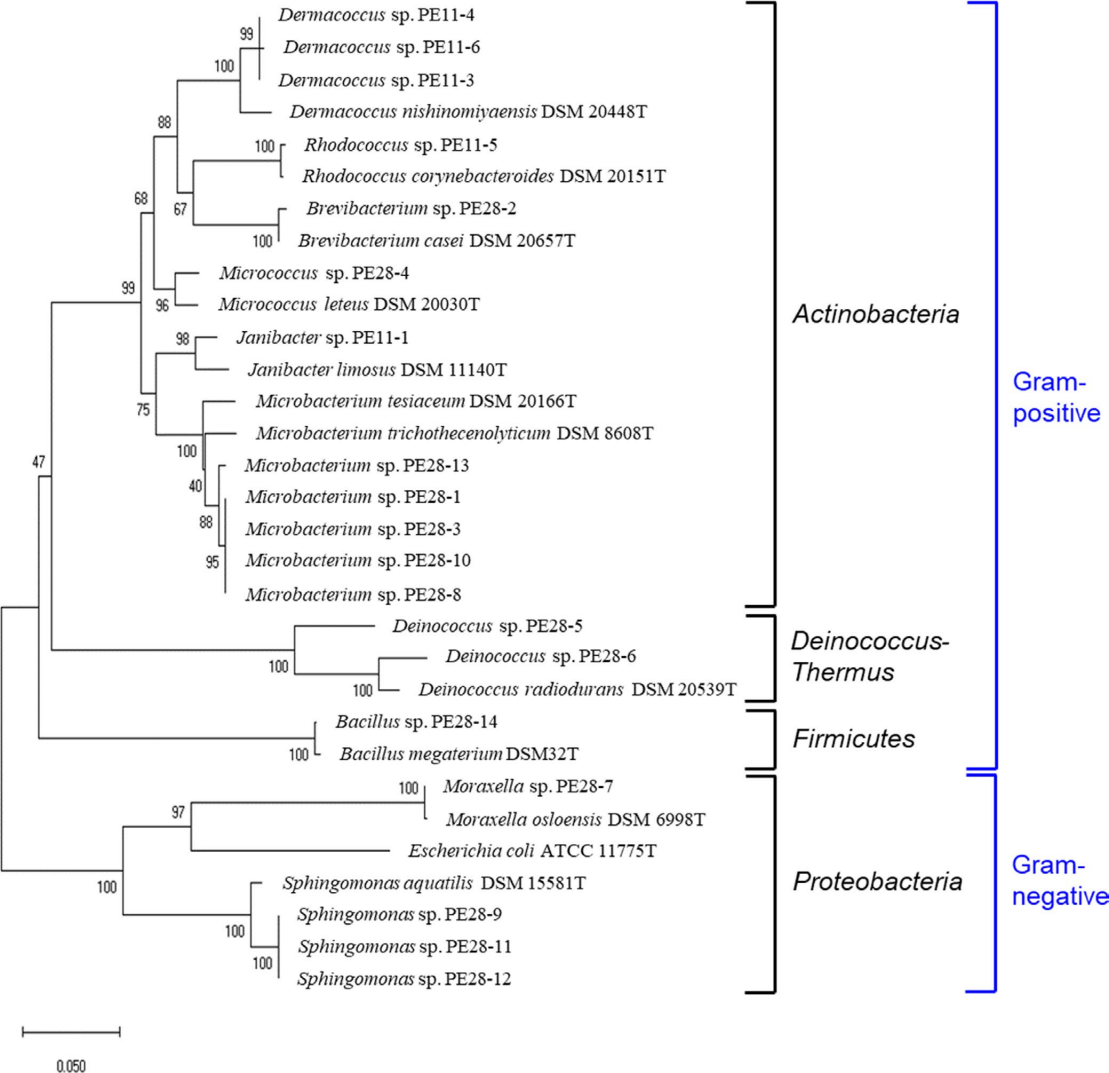
Phylogenetic relationships of bacterial strains recovered from the emerging seedlings from *P*. *edulis* seeds, based on the 16S rDNA sequence. Bootstrap values from 1000 replications are shown at each of the branch points on the tree

Endophytes were isolated from the seedlings emerging from seeds of *P*. *edulis* grown in Japan. Seeds were collected from the fruit and surface‐sterilized, as described in the Materials and methods. After surface‐sterilization, the seeds were cut and incubated on agar medium at 30ºC for approximately 1 month. However, no colonies appeared on the plates. Attempts to isolate microorganisms from the homogenized seeds were also unsuccessful. As we observed that the cut seeds sprouted after incubation, the resulting seedlings were aseptically sectioned into small fragments and further incubated on the plates (Figure 1). Interestingly, microbial colonies were generated from the seedlings derived from the seeds under sterile conditions. Using this isolation procedure, a total of 19 bacterial strains were isolated, of which 5 and 14 strains were derived from seeds of *P*. *edulis* grown in Okinawa and Kumamoto Prefectures, respectively (Table 1). The isolated strains were taxonomically identified based on 16S rDNA sequencing, and a phylogenetic tree of the resulting sequences was constructed (Figure 2). The isolated bacteria belonged to 10 different genera: *Dermacoccus*, *Rhodococcus*, *Brevibacterium*, *Micrococcus*, *Janibacter*, *Microbacterium*, *Deinococcus*, *Bacillus*, *Moraxella*, and *Sphingomonas* (Figure 2 and Table 1). Three strains (PE11‐1, PE28‐5, and PE28‐6) exhibited low identity (<97%) to previously reported sequences of typical strains, suggesting that these strains constitute new genera or species. Intriguingly, 15 of the strains were classified as Gram‐positive, and 12 of these Gram‐positive strains belonged to 1 phylum, *Actinobacteria* (Figure 2).

### 3.2 Effect of piceatannol on the growth of the isolated bacteria

**FIGURE A1:**
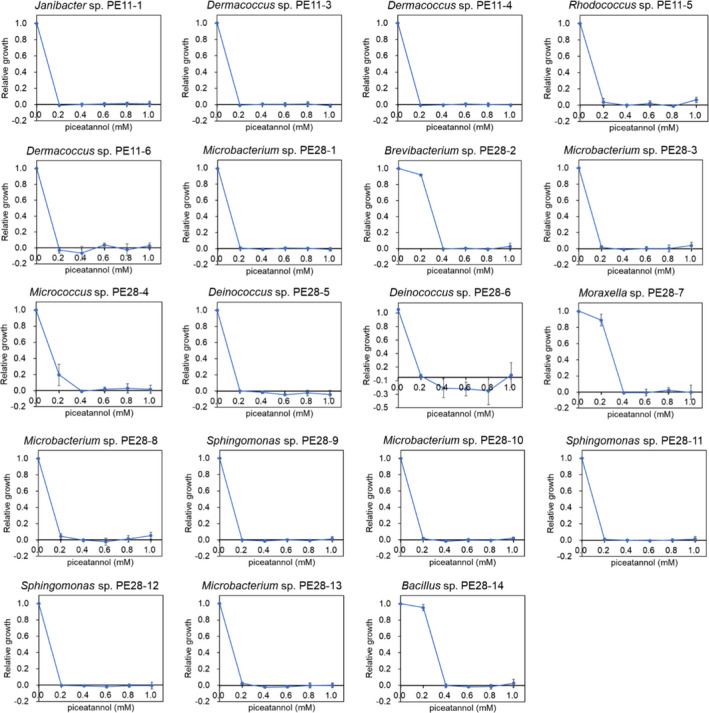
Effect of piceatannol on the growth of the isolated bacteria. Each bacterium was inoculated into the LB medium containing piceatannol (0–1.0 mM) and further cultivated at 30℃ for 2 days. After cultivation, bacterial growth was determined by measuring the OD_600_. The average value of the culture without piceatannol was expressed as 1.0. Data are the average of three independent experiments, and error bars indicate the standard deviation of the mean

Because the bacteria were isolated from the emerging seedlings from *P*. *edulis* seeds containing piceatannol at extremely high levels as a defense compound, we investigated the effect of this compound on the growth of the isolated bacteria by determining the MIC and MBC values, as described in the Materials and methods. Strains PE28‐2, PE28‐4, PE28‐7, PE28‐8, and PE28‐14 grew in liquid LB medium containing 0.2 mM (49 µg/mL) piceatannol, whereas 0.4 mM piceatannol inhibited their growth (MIC 0.4 mM) (Table 2 and Figure A1). The other strains did not grow even in the presence of 0.2 mM piceatannol (MIC < 0.2 mM) (Table 2 and Figure A1). These results indicate that piceatannol strongly inhibits the growth of the isolated bacteria.

**TABLE 2:** Antibacterial activity of piceatannol against the bacteria isolated from the emerging seedlings from *P*. *edulis* seeds

Strain	MIC (mM)^a^	MBC (mM)^b^
PE11‐1	< 0.2	0.4
PE11‐3	< 0.2	0.4
PE11‐4	< 0.2	0.4
PE11‐5	< 0.2	0.8
PE11‐6	< 0.2	< 0.2
PE28‐1	< 0.2	0.6
PE28‐2	0.4	0.8
PE28‐3	< 0.2	0.4
PE28‐4	0.4	0.8
PE28‐5	< 0.2	< 0.2
PE28‐6	< 0.2	< 0.2
PE28‐7	0.4	0.8
PE28‐8	0.4	0.6
PE28‐9	< 0.2	0.4
PE28‐10	< 0.2	0.4
PE28‐11	< 0.2	0.6
PE28‐12	< 0.2	0.6
PE28‐13	< 0.2	0.6
PE28‐14	0.4	0.6

^a^ Minimal inhibitory concentration (MIC) was determined based on the results in Figure A1 as described in the Materials and methods.

^b^ The minimal bactericidal concentration (MBC) was determined by visual inspection as described in the Materials and methods.

In contrast, after bacteria were cultured in liquid LB medium containing piceatannol at concentrations higher than the MIC, all strains except PE11‐6, PE28‐5, and PE28‐6 grew on solid LB medium lacking piceatannol (Table 2). In particular, *Rhodococcus* sp. PE11‐5, *Brevibacterium* sp. PE28‐2, *Moraxella* sp. PE28‐4, and *Bacillus* sp. PE28‐7 grew on agar medium after treatment with 0.6 mM piceatannol (MBC 0.8 mM), although this concentration of piceatannol strongly inhibited the growth of these 4 strains (Table 2). These results indicate that piceatannol exerts bacteriostatic rather than bactericidal effects on most of the isolated bacteria.

### 3.3 Growth of the isolated bacteria on different carbon sources

We also examined the growth of the isolated bacteria on different carbon sources. Resveratrol and piceatannol were examined first, as these compounds are present in the seeds. Bacteria were cultivated for 7 days in KG medium supplemented with resveratrol or piceatannol as a carbon source. However, no bacteria grew on a medium containing either resveratrol or piceatannol. Glucose and 4‐hydroxyphenylacetic acid were also tested as typical carbohydrate and aromatic compounds, respectively, that are assimilated by many species of bacteria. All strains grew in medium supplemented with glucose (OD_600_ > 0.1, Table 3). *Janibacter* sp. PE11‐1, *Brevibacterium* sp. PE28‐2, and *Microbacterium* sp. PE28‐13 grew in medium supplemented with 4‐hydroxyphenylacetic acid (OD_600_ > 0.1, Table 3). These results indicate that all of the isolated bacteria readily assimilate glucose but not resveratrol or piceatannol. In addition, 3 of the 19 strains could utilize 4‐hydroxyphenylacetic acid as a carbon source.

**TABLE 3:** Growth of the isolated bacteria on glucose and 4‐hydroxyphenylacetic acid

Strain	Growth (OD_600_)^a^	
	Glucose	4‐hydroxyphenylacetic acid
PE11‐1	0.18 ± 0.01	0.09 ± 0.02
PE11‐3	0.67 ± 0.07	0.02 ± 0.01
PE11‐4	0.44 ± 0.06	0.01 ± 0.01
PE11‐5	0.50 ± 0.01	0.01 ± 0.01
PE11‐6	0.21 ± 0.14	0.00 ± 0.01
PE28‐1	0.48 ± 0.20	−0.02 ± 0.01
PE28‐2	0.29 ± 0.10	0.42 ± 0.01
PE28‐3	0.15 ± 0.04	−0.01 ± 0.02
PE28‐4	0.44 ± 0.12	0.02 ± 0.04
PE28‐5	0.37 ± 0.08	−0.01 ± 0.03
PE28‐6	0.18 ± 0.03	0.00 ± 0.01
PE28‐7	0.28 ± 0.02	0.01 ± 0.00
PE28‐8	0.32 ± 0.02	−0.01 ± 0.02
PE28‐9	0.43 ± 0.17	−0.01 ± 0.01
PE28‐10	0.42 ± 0.11	−0.01 ± 0.01
PE28‐11	0.07 ± 0.01	−0.01 ± 0.01
PE28‐12	0.39 ± 0.14	0.00 ± 0.01
PE28‐13	0.42 ± 0.04	0.33 ± 0.03
PE28‐14	0.37 ± 0.01	−0.01 ± 0.01

^a^ The isolated bacteria were cultivated in a medium containing glucose or 4‐hydroxyphenylacetic acid as a carbon source (5 mM) for 7 days. Data are the average of three independent experiments, and error bars indicate the standard deviation of the mean.

### 3.4 Biocatalytic activity of the isolated bacteria toward resveratrol and piceatannol

**FIGURE 3:**
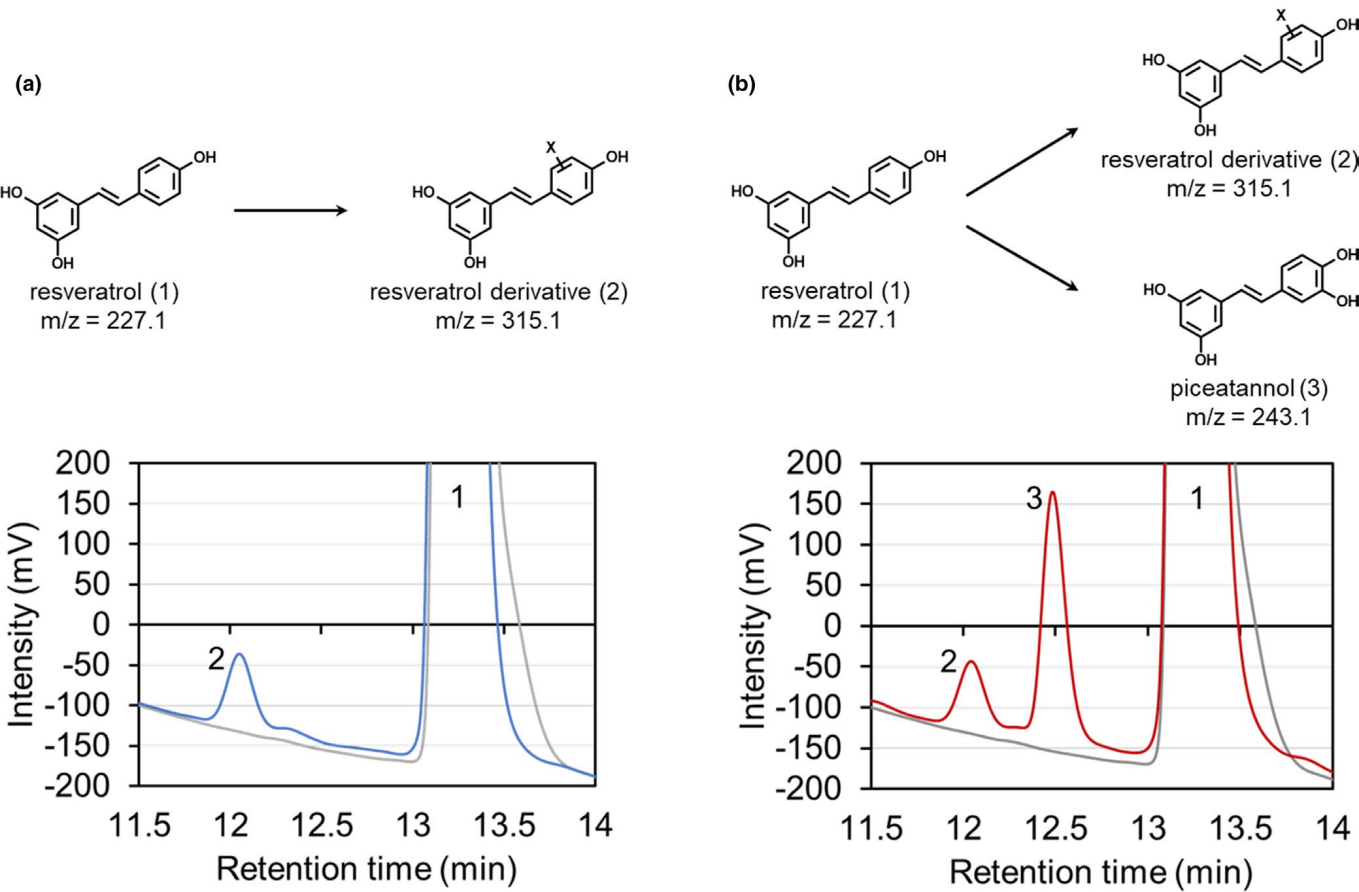
HPLC analysis of reactions of *Brevibacterium* sp. PE28‐2 with resveratrol. PE28‐2 cells grown on a medium containing glucose (a) or 4‐hydroxyphenylacetic acid (b) were incubated with resveratrol. Peaks 1 (at 13.4 min), 2 (at 12.0 min), and 3 (at 12.4 min) corresponded to resveratrol, a resveratrol derivative, and piceatannol, respectively. HPLC chromatograms for the control reaction using autoclaved cells are shown by gray lines. “X” on the chemical structure indicates an unidentified moiety corresponding to *m*/*z* = 88.0

**FIGURE A2:**
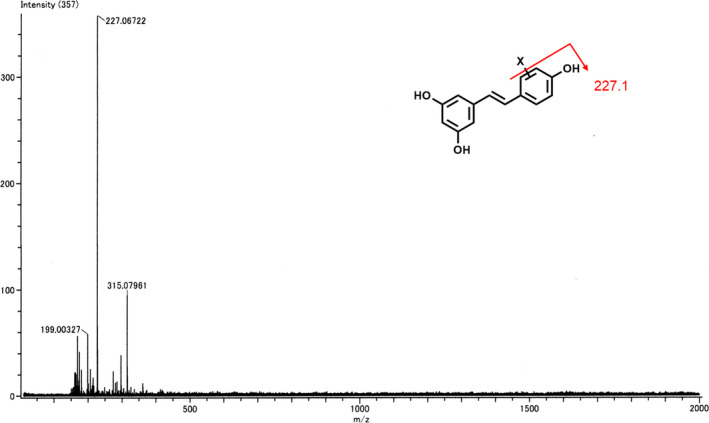
LC‐MS analysis of the reaction product formed from resveratrol by *Brevibacterium* sp. PE28‐2. LC‐MS spectrum for peak 2 in Figure 3 is shown. The reaction product (m/z = 315.1) was identified as a resveratrol derivative with a modification (m/z = 88.0) on the resveratrol moiety (m/z = 227.1)

**FIGURE A3:**
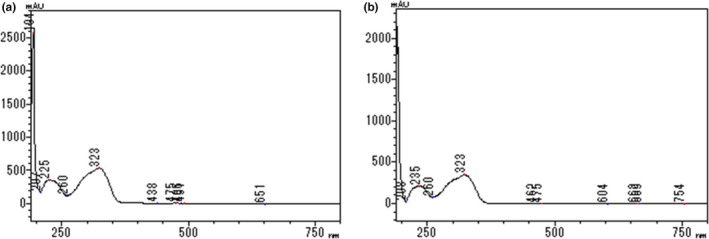
UV‐visible absorption analysis of the reaction product formed from resveratrol by *Brevibacterium* sp. PE28‐2. The UV‐visible absorption spectrum for peak 3 in Figure 3 is shown in (a). The spectrum coincided with that of an authentic sample of piceatannol (b)

We next explored the biocatalytic potential of the isolated bacteria to convert resveratrol and piceatannol to other stilbene derivatives. Bacteria were cultivated for 7 days on carbon sources that the strains could assimilate. The bacteria were then harvested and incubated for 24 h in a buffer solution containing 5 mM resveratrol or 2 mM piceatannol. Although most of the strains did not exhibit any biocatalytic activity, *Brevibacterium* sp. PE28‐2 did convert resveratrol. HPLC analysis of the reaction of glucose‐grown PE28‐2 cells with resveratrol showed a product peak (retention time, 12.0 min) in addition to the substrate peak (13.4 min) (Figure 3a). This product was not detected in the control reaction using autoclaved PE28‐2 cells. Furthermore, LC‐MS analysis revealed that the [M‐H]^−^ ion of this product was *m*/*z* = 315.1 (Figure A2). In addition to the parent ion, the [M‐H]^−^ ion of the resveratrol moiety (*m*/*z* = 227.1) was detected (Figure A2). These results suggest that strain PE28‐2 converts resveratrol to a previously unknown resveratrol derivative with a modification corresponding to *m*/*z* = 88.0 (Figure 3a). Interestingly, when cells grown on 4‐hydroxyphenylacetic acid were incubated with resveratrol, HPLC analysis showed an additional product peak (retention time, 12.4 min) (Figure 3b). The compound corresponding to this peak was identified as a monooxygenation product of resveratrol based on the determination of its mass ([M‐H]^−^ *m*/*z* = 243.1). Furthermore, the retention time and UV‐visible absorption spectrum of this product were consistent with those of an authentic sample of piceatannol (Figure A3). Based on these observations, this product was identified as piceatannol (Figure 3b).

**FIGURE 4:**
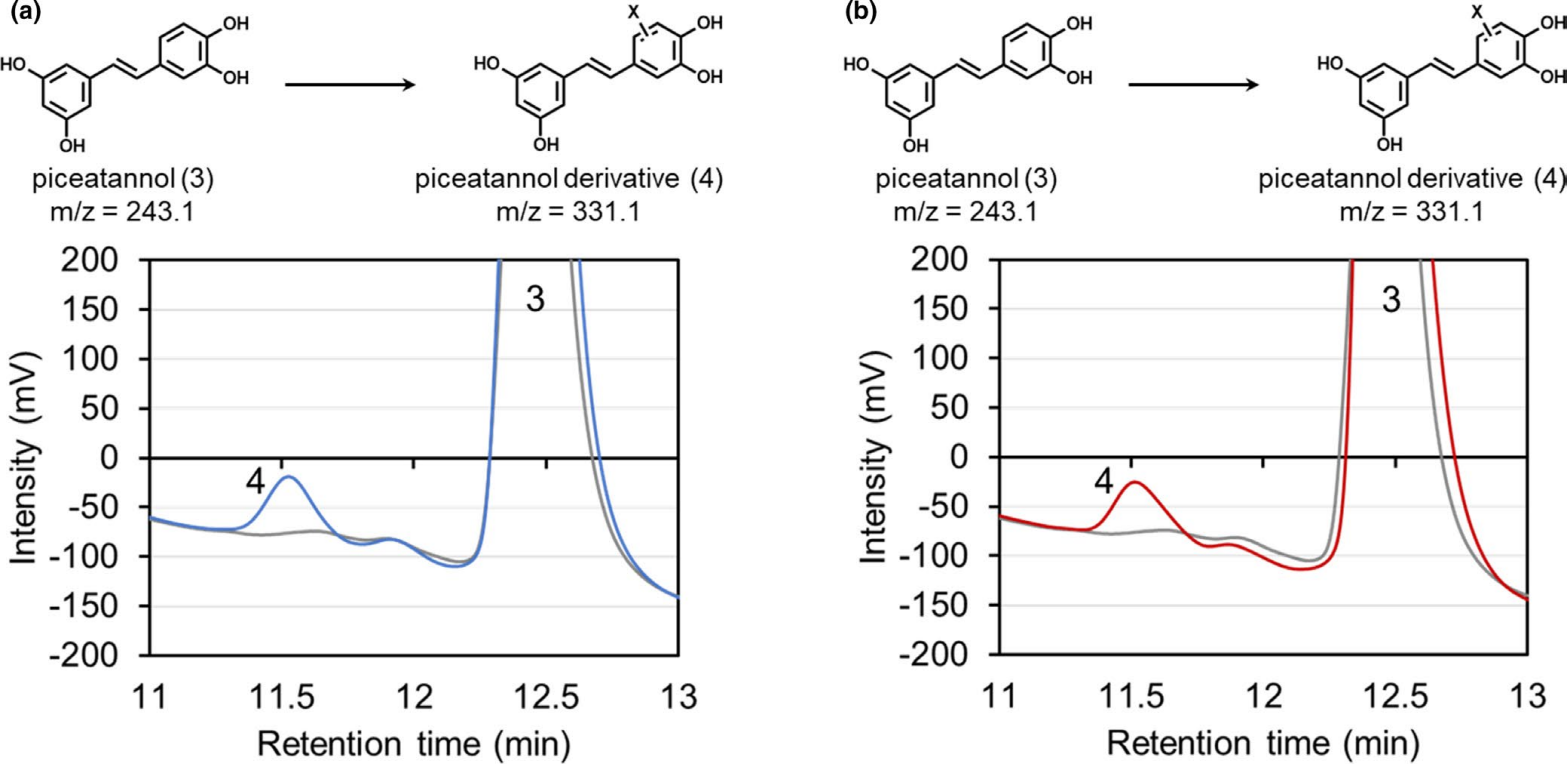
HPLC analysis of reactions of *Brevibacterium* sp. PE28‐2 with piceatannol. PE28‐2 cells grown on a medium containing glucose (a) or 4‐hydroxyphenylacetic acid (b) were incubated with piceatannol. Peaks 3 (at 12.4 min) and 4 (at 11.5 min) corresponded to piceatannol and a piceatannol derivative, respectively. HPLC chromatograms for the control reaction using autoclaved cells are shown by gray lines. “X” on the chemical structure indicates an unidentified moiety corresponding to *m*/*z* = 88.0

**FIGURE A4:**
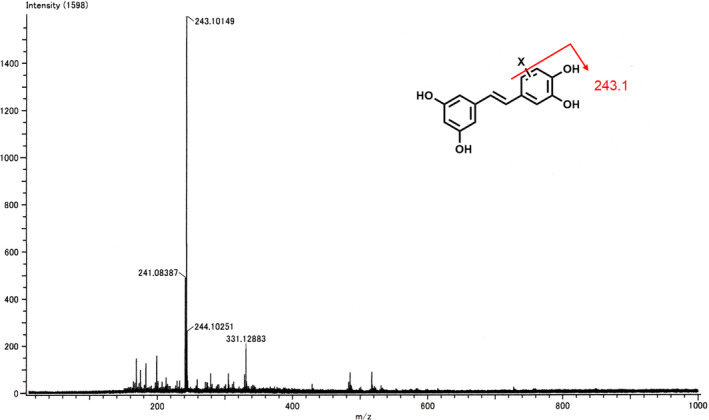
LC‐MS analysis of the reaction product formed from piceatannol by *Brevibacterium* sp. PE28‐2. LC‐MS spectrum for peak 4 in Figure 4 is shown. The reaction product (m/z = 331.1) was identified as a piceatannol derivative with a modification (m/z = 88.0) on the piceatannol moiety (m/z = 243.1)

We also found that *Brevibacterium* sp. PE28‐2 converted piceatannol. HPLC analysis of the reaction of glucose‐grown PE28‐2 cells with piceatannol, and also the reaction of cells grown on 4‐hydroxyphenylacetic acid with piceatannol, showed a product peak (retention time, 11.5 min) in addition to the substrate peak (12.4 min) (Figure 4). LC‐MS analysis revealed the parent [M‐H]^−^ ion (*m*/*z* = 331.1) and [M‐H]^−^ ion of the piceatannol moiety (*m*/*z* = 243.1) (Figure A4). These results suggest that strain PE28‐2 converts piceatannol to a previously unknown piceatannol derivative with a modification corresponding to *m*/*z* = 88.0 (Figure 4), in the same manner as the conversion of resveratrol described above.

## 4 DISCUSSION

Although the human placenta and fetus are free of microorganisms (Segata, 2019), these organisms can reside inside plant seeds. Endophytic bacteria enter various plant compartments, including roots, stems, and leaves, from the external environment and colonize the intercellular spaces. Such colonization reportedly requires that the microorganisms exhibit motility, adhesion, and cell wall degradation activity (Liu et al., 2017; Reinhold‐Hurek & Hurek, 2011). Furthermore, some strains of bacteria can be transported from the vegetative parts of the plant to the seeds via xylem vessels (Frank et al., 2017). Growing evidence also suggests that seed endophytes are transmitted from generation to generation (Frank et al., 2017). Inhabiting seeds could be beneficial for bacteria because it would enable them to promptly colonize new generations of plants where appropriate nutrients are available. This situation is often advantageous to plants because they can select bacteria that are helpful for germination, seedling development, and plant growth (Shahzad et al., 2018; Truyens et al., 2015). To date, bacteria have been detected inside surface‐sterilized seeds of a variety of plants (Frank et al., 2017; Shahzad et al., 2018; Truyens et al., 2015). However, the isolation of seed endophytes remains challenging due to the unusual habitat.

In this study, we successfully isolated several bacterial strains from the seedlings emerging from *P*. *edulis* seeds. To our knowledge, this is the first report describing seed endophytes of *P*. *edulis*. Although no microorganisms grew from surface‐sterilized seeds, bacterial colonies were generated from seedlings derived from the seeds under sterile conditions. Endophytes can generally form colonies on agar growth medium directly from seeds (Compant et al., 2011; Vega et al., 2005; Verma et al., 2017). One possible explanation for this phenomenon is that inhibitors in the seeds such as piceatannol hamper the growth of bacteria. Transmission from seeds to seedlings might allow the bacteria to escape from the inhibitors, thus enabling them to grow on an agar medium. A phenomenon similar to that proposed in this hypothesis was observed during the isolation of bacteria from wheat seeds (Robinson et al., 2016). That study suggested that antimicrobial puroindolines and phenolic acids in wheat seeds inhibit bacterial growth. The isolation procedure described here (Figure 1) should be generally applicable to the isolation of culturable bacteria from various seeds, particularly those containing antimicrobial compounds at high levels.

Using our isolation procedure, a total of 19 bacterial strains were isolated from the emerging seedlings from *P*. *edulis* seeds (Figure 2 and Table 1), of which 15 strains were classified as Gram‐positive. The Gram‐positive strains belonged to 3 phyla, *Actinobacteria* (12 strains), *Deinococcus*‐*Thermus* (2 strains), and *Firmicutes* (1 strain). The remaining 4 strains were classified as Gram‐negative and belonged to 1 phylum, *Proteobacteria*. Bacteria of *Actinobacteria*, *Firmicutes*, and *Proteobacteria* have frequently been recovered from plant seeds, but there have been no reports to date concerning culturable *Deinococcus*‐*Thermus* seed endophytes (Shahzad et al., 2018; Truyens et al., 2015). Notably, Gram‐positive bacteria dominated the isolated microorganisms. During the maturation process, seeds lose water, leading to an increase in osmotic pressure in the seeds. Gram‐positive bacteria possess a thicker peptidoglycan cell wall layer than Gram‐negative bacteria, which may render them more resistant to high osmotic pressure (Naylor & Coleman‐Derr, 2018). In addition, 1 *Bacillus* strain belonging to *Firmicutes* was isolated, which can form endospores to resist environmental stress. Furthermore, we also isolated 2 *Deinococcus* strains that are reportedly resistant to dehydration (Bauermeister et al., 2011; Mattimore & Battista, 1996). Similar to our results, it was reported that more Gram‐positive isolates can be cultured from rice seeds as the seeds mature (Mano et al., 2007). To clarify the reason for the predominance of Gram‐positive bacteria, more detailed characterizations, including isolation of bacteria from seeds at different stages, as well as metagenomic analyses, are needed.

Different genera of bacteria were isolated depending on where the plants were grown (Okinawa Prefecture versus Kumamoto Prefecture) (Table 1). These results suggest that the symbiotic relationship between *P*. *edulis* plants and the bacteria we isolated is not obligate, but the seeds might select specific microorganisms suitable for the seed environment via the vegetative parts from among the microbial communities around the plants.

We found that piceatannol inhibited the growth of all isolated bacteria, with MIC values of <0.4 mM (Table 2 and Figure A1). This concentration corresponds to 0.10 mg/g, which is lower than the concentration of piceatannol in *P*. *edulis* seeds (ca. 2 mg/g). Although it is difficult to compare concentrations of this compound between the liquid medium and seeds, these results suggest that piceatannol in the seeds inhibits the growth of these bacteria in situ. In contrast, most of the isolated bacteria grew on a solid medium after treatment with piceatannol at concentrations higher than the MIC (MBC > 0.4 mM) (Table 2). Thus, it is conceivable that the effect of piceatannol on these bacteria is bacteriostatic rather than bactericidal. These results also support the possibility that static bacteria in seeds are transmitted to seedlings during the germination process, in which the bacteria are not exposed to defense compounds such as piceatannol. As a consequence, these bacteria may be able to establish colonies from the seedlings on the plates. In other words, bacteria capable of enduring a high concentration of piceatannol and high osmotic pressure can survive in seeds.

We also explored the biocatalytic potential of the isolated bacteria to convert resveratrol and piceatannol. No isolated bacteria assimilated resveratrol or piceatannol, but strains PE11‐1, PE28‐2, and PE28‐13 utilized 4‐hydroxybenzoic acid as a carbon source (Table 3). In addition, interestingly, *Brevibacterium* sp. PE28‐2 hydroxylated resveratrol to piceatannol (Figures 3 and A3). Strain PE28‐2 is the first endophyte shown to produce piceatannol, although several bacteria isolated from soils reportedly exhibit such activity (Furuya et al., 2019). It is conceivable that this bacterium evolved its metabolic system due to exposure to aromatic compounds such as resveratrol and piceatannol in seeds and other environments. The resveratrol‐hydroxylating activity of strain PE28‐2 was induced when the bacteria were cultivated on 4‐hydroxyphenylacetic acid (Figure 3), which is reportedly contained in many plants (Kindl, 1969). Thus, 4‐hydroxyphenylacetic acid or its analogs present in *P*. *edulis* might induce the hydroxylation activity in situ. We also found that glucose‐grown PE28‐2 cells converted resveratrol and piceatannol to their novel respective derivatives, which have a modification corresponding to *m*/*z* = 88.0 (e.g., C_3_H_4_O_3_) (Figures 3, 4, A2 and A4). Because attempts to identify the products were unsuccessful due to low yield, further examinations of reaction conditions are needed to enhance the production of these compounds. *Brevibacterium* sp. PE28‐2 could be a useful biocatalyst for the production of piceatannol and novel stilbene derivatives.

## 5 ETHICS STATEMENT

None required.

## 6 CONFLICT OF INTEREST

None declared.

## AUTHOR CONTRIBUTIONS

**Aoi Ishida:** Conceptualization (supporting); Data curation (lead); Formal analysis (lead); Investigation (equal); Methodology (equal); Resources (equal); Software (equal); Validation (equal); Visualization (equal); Writing‐original draft (supporting); Writing‐review & editing (equal). **Toshiki Furuya:** Conceptualization (lead); Data curation (supporting); Formal analysis (supporting); Funding acquisition (lead); Investigation (equal); Methodology (equal); Project administration (lead); Resources (equal); Software (equal); Supervision (lead); Validation (equal); Visualization (equal); Writing‐original draft (lead); Writing‐review & editing (equal).

## Data Availability

All data generated or analyzed during this study are included in this article. The sequence data of the isolated bacteria are available in GenBank at https://www.ncbi.nlm.nih.gov/genbankunder accession numbers listed in Table 1.

## References

[mbo31226-bib-0001] Bauermeister, A., Moeller, R., Reitz, G., Sommer, S., & Rettberg, P. (2011). Effect of relative humidity on *Deinococcus radiodurans*’ resistance to prolonged desiccation, heat, ionizing, germicidal, and environmentally relevant UV radiation. Microbial Ecology, 61, 715–722. 10.1007/s00248-010-9785-4 21161207

[mbo31226-bib-0002] Brader, G., Compant, S., Mitter, B., Trognitz, F., & Sessitsch, A. (2014). Metabolic potential of endophytic bacteria. Current Opinion in Biotechnology, 27, 30–37. 10.1016/j.copbio.2013.09.012 24863894 PMC4045207

[mbo31226-bib-0003] Compant, S., Mitter, B., Colli‐Mull, J. G., Gangl, H., & Sessitsch, A. (2011). Endophytes of grapevine flowers, berries, and seeds: identification of cultivable bacteria, comparison with other plant parts, and visualization of niches of colonization. Microbial Ecology, 62, 188–197. 10.1007/s00248-011-9883-y 21625971

[mbo31226-bib-0004] Frank, A. C., Saldierna Guzmán, J. P., & Shay, J. E. (2017). Transmission of Bacterial Endophytes. Microorganisms, 5(4), 70. 10.3390/microorganisms5040070 PMC5748579 29125552

[mbo31226-bib-0005] Furuya, T., Hirose, S., Osanai, H., Semba, H., & Kino, K. (2011). Identification of the monooxygenase gene clusters responsible for the regioselective oxidation of phenol to hydroquinone in mycobacteria. Applied and Environmental Microbiology, 77, 1214–1220. 10.1128/AEM.02316-10 21183637 PMC3067212

[mbo31226-bib-0006] Furuya, T., Imaki, N., Shigei, K., Sai, M., & Kino, K. (2019). Isolation and characterization of Gram‐negative and Gram‐positive bacteria capable of producing piceatannol from resveratrol. Applied Microbiology and Biotechnology, 103, 5811–5820. 10.1007/s00253-019-09875-z 31093702

[mbo31226-bib-0007] Furuya, T., & Kino, K. (2014). Regioselective synthesis of piceatannol from resveratrol: catalysis by two‐component flavin‐dependent monooxygenase HpaBC in whole cells. Tetrahedron Letters, 55, 2853–2855. 10.1016/j.tetlet.2014.03.076

[mbo31226-bib-0008] Hashimoto, T., Nozawa, D., Mukai, K., Matsuyama, A., Kuramochi, K., & Furuya, T. (2019). Monooxygenase‐catalyzed regioselective hydroxylation for the synthesis of hydroxyequols. RSC Advances, 9, 21826–21830. 10.1039/C9RA03913A PMC9066559 35518870

[mbo31226-bib-0009] Jeandet, P., Vannozzi, A., Sobarzo‐Sánchez, E., Uddin, M. S., Bru, R., Martínez‐Márquez, A., Clément, C., Cordelier, S., Manayi, A., Nabavi, S. F., Rasekhian, M., El‐Saber Batiha, G., Khan, H., Morkunas, I., Belwal, T., Jiang, J., Koffas, M., & Nabavi, S. M. (2021). Phytostilbenes as agrochemicals: biosynthesis, bioactivity, metabolic engineering and biotechnology. Natural Product Reports, 38, 1282–1329. 10.1039/D0NP00030B 33351014

[mbo31226-bib-0010] Kindl, H. (1969). Biosynthesis and metabolism of hydroxyphenylacetic acids in higher plants. European Journal of Biochemistry, 7, 340–347. 10.1111/j.1432-1033.1969.tb19614.x 5791579

[mbo31226-bib-0011] Kurokawa, M., Nakano, M., Kitahata, N., Kuchitsu, K., & Furuya, T. (2021). An efficient direct screening system for microorganisms that activate plant immune responses based on plant‐microbe interactions using cultured plant cells. Scientific Reports, 11, 7396. 10.1038/s41598-021-86560-0 33795728 PMC8016971

[mbo31226-bib-0012] Kusakabe, Y., Mizutani, S., Kamo, S., Yoshimoto, T., Tomoshige, S., Kawasaki, T., Takasawa, R., Tsubaki, K., & Kuramochi, K. (2019). Synthesis, antibacterial and cytotoxic evaluation of flavipucine and its derivatives. Bioorganic & Medicinal Chemistry Letters, 29, 1390–1394. 10.1016/j.bmcl.2019.03.034 30935798

[mbo31226-bib-0013] Liu, H., Carvalhais, L. C., Crawford, M., Singh, E., Dennis, P. G., Pieterse, C. M. J., & Schenk, P. M. (2017). Inner plant values: diversity, colonization and benefits from endophytic bacteria. Frontiers in Microbiology, 19, 2552. 10.3389/fmicb.2017.02552 PMC5742157 29312235

[mbo31226-bib-0014] Majeed, A., Majeed, M., Thajuddin, N., Arumugam, S., Ali, F., Beede, K., Adams, S. J., & Gnanamani, M. (2019). Bioconversion of curcumin into calebin‐A by the endophytic fungus *Ovatospora brasiliensis* EPE‐10 MTCC 25236 associated with *Curcuma caesia* . AMB Express, 9, 79. 10.1186/s13568-019-0802-9 31144200 PMC6541684

[mbo31226-bib-0015] Mano, H., Tanaka, F., Nakamura, C., Kaga, H., & Morisaki, H. (2007). Culturable endophytic bacterial flora of the maturing leaves and roots of rice plants (*Oryza sativa*) cultivated in a paddy field. Microbes and Environments., 22, 175–185. 10.1264/jsme2.22.175

[mbo31226-bib-0016] Matsui, Y., Sugiyama, K., Kamei, M., Takahashi, T., Suzuki, T., Katagata, Y., & Ito, T. (2010). Extract of passion fruit (*Passiflora edulis*) seed containing high amounts of piceatannol inhibits melanogenesis and promotes collagen synthesis. Journal of Agricultural and Food Chemistry, 58, 11112–11118. 10.1021/jf102650d 20822151

[mbo31226-bib-0017] Mattimore, V., & Battista, J. R. (1996). Radioresistance of *Deinococcus radiodurans*: functions necessary to survive ionizing radiation are also necessary to survive prolonged desiccation. Journal of Bacteriology, 178, 633–637. 10.1128/jb.178.3.633-637.1996 8550493 PMC177705

[mbo31226-bib-0018] Naylor, D., & Coleman‐Derr, D. (2018). Drought stress and root‐associated bacterial communities. Frontiers in Plant Science, 8, 2223. 10.3389/fpls.2017.02223 29375600 PMC5767233

[mbo31226-bib-0019] Piotrowska, H., Kucinska, M., & Murias, M. (2012). Biological activity of piceatannol: leaving the shadow of resveratrol. Mutation Research, 750, 60–82. 10.1016/j.mrrev.2011.11.001 22108298

[mbo31226-bib-0020] Rathnayake, G. R. N., Kumar, N. S., Jayasinghe, L., Araya, H., & Fujimoto, Y. (2018). Chemical investigation of metabolites produced by an endophytic fungi *Phialemonium curvatum* from the leaves of *Passiflora edulis* . Natural Product Research, 32, 2483–2486. 10.1080/14786419.2017.1416373 29260908

[mbo31226-bib-0021] Reinhold‐Hurek, B., & Hurek, T. (2011). Living inside plants: bacterial endophytes. Current Opinion in Plant Biology, 14, 435–443. 10.1016/j.pbi.2011.04.004 21536480

[mbo31226-bib-0022] Robinson, R. J., Fraaije, B. A., Clark, I. M., Jackson, R. W., Hirsch, P. R., & Mauchline, T. H. (2016). Wheat seed embryo excision enables the creation of axenic seedlings and Koch's postulates testing of putative bacterial endophytes. Scientific Reports, 6, 25581. 10.1038/srep25581 27151146 PMC4858700

[mbo31226-bib-0023] Rodriguez, P., Gonzalez, D., & Rodríguez Giordano, S. (2016). Endophytic microorganisms: A source of potentially useful biocatalysts. Journal of Molecular Catalysis. B, Enzymatic, 133, S569–S581. 10.1016/j.molcatb.2017.02.013

[mbo31226-bib-0024] Santos, M. S., Orlandelli, R. C., Polonio, J. C., dos Santos Ribeiro, M. A., Sarragiotto, M. H., Azevedo, J. L., & Pamphile, J. A. (2017). Endophytes isolated from passion fruit plants: molecular identification, chemical characterization and antibacterial activity of secondary metabolites. Journal of Applied Pharmaceutical Science, 7(04), 038–043.

[mbo31226-bib-0025] Segata, N. (2019). No bacteria found in healthy placentas. Nature, 572, 317–318. 10.1038/d41586-019-02262-8 31406307

[mbo31226-bib-0026] Seyed, M. A., Jantan, I., Bukhari, S. N., & Vijayaraghavan, K. (2016). A Comprehensive review on the chemotherapeutic potential of piceatannol for cancer treatment, with mechanistic insights. Journal of Agricultural and Food Chemistry, 64, 725–737. 10.1021/acs.jafc.5b05993 26758628

[mbo31226-bib-0027] Shahzad, R., Khan, A. L., Bilal, S., Asaf, S., & Lee, I. J. (2018). What is there in seeds? Vertically transmitted endophytic resources for sustainable improvement in plant growth. Frontiers in Plant Science, 9, 24. 10.3389/fpls.2018.00024 29410675 PMC5787091

[mbo31226-bib-0028] Truyens, S., Weyens, N., Cuypers, A., & Vangronsveld, J. (2015). Bacterial seed endophytes: genera, vertical transmission and interaction with plants. Environmental Microbiology Reports, 7, 40–50. 10.1111/1758-2229.12181

[mbo31226-bib-0029] Vega, F. E., Pava‐Ripoll, M., Posada, F., & Buyer, J. S. (2005). Endophytic bacteria in *Coffea arabica* L. Journal of Basic Microbiology, 45, 371–380. 10.1002/jobm.200410551 16187260

[mbo31226-bib-0030] Verma, S. K., Kingsley, K., Irizarry, I., Bergen, M., Kharwar, R. N., & White, J. F. Jr (2017). Seed‐vectored endophytic bacteria modulate development of rice seedlings. Journal of Applied Microbiology, 122, 1680–1691. 10.1111/jam.13463 28375579

[mbo31226-bib-0031] Zhou, X., Zhu, H., Liu, L., Lin, J., & Tang, K. (2010). A review: recent advances and future prospects of taxol‐producing endophytic fungi. Applied Microbiology and Biotechnology, 86, 1707–1717. 10.1007/s00253-010-2546-y 20358192

